# Robust disturbance-aware actor–critic reinforcement learning for multi-DOF robotic manipulators

**DOI:** 10.1371/journal.pone.0354740

**Published:** 2026-10-06

**Authors:** Viet Ngu Nguyen, Thi Minh Tam Le, Duc Hung Pham, Huu Cuong Nguyen

**Affiliations:** Faculty of Electrical and Electronic Engineering, Hung Yen University of Technology and Education, Vietnam; Southeast University, CHINA

## Abstract

Building on recent insights that augmenting reinforcement‐learning policies with disturbance estimates improves robustness and sim-to-real transfer, this paper proposes a disturbance-aware actor–critic RL framework for high‐precision robotic manipulators. We derive the dynamics of manipulators ranging from two to six degrees of freedom and design a nonlinear disturbance observer that provides real-time estimates of lumped uncertainties. Unlike conventional feedforward-only designs, the proposed DACRL framework deeply integrates DOB information into the actor-critic’s learning state, cost function, and update laws to explicitly compensate for unknown dynamics and disturbances. A Lyapunov-based analysis proves that tracking errors, observer errors and neural-network weight errors remain uniformly ultimately bounded. Extensive simulations show that the proposed controller achieves sub-degree tracking errors across multiple DOF cases, with the 2-DOF example achieving steady-state errors below ±0.02 rad (Joint 1) and ±0.05 rad (Joint 2) and delivering faster convergence and smoother torques than conventional RL and DOB baselines. The disturbance-aware controller generalizes across trajectories and payloads, offering improved robustness while retaining learning flexibility. While the current study focuses on performance and robustness, future work could explore the integration of disturbance-observer-based control barrier functions to formally address safety constraints during the learning process. Simulation results suggest that the disturbance-aware controller can improve robustness while retaining learning flexibility. Real-platform validation remains an important direction for future work.

## 1. Introduction

Reinforcement learning (RL) has been increasingly adopted in robotics as a data-driven paradigm for sequential decision-making and control in complex nonlinear systems [1]. Broad overviews of RL algorithms and methodological families further consolidate this trend and clarify common design choices [2]. Recent monographs also provide rigorous foundations for sequential decision-making and optimal control perspectives that are frequently used in continuous-time robotic control formulations [3].

Within this context, model-based RL has attracted growing attention due to its potential for improved sample efficiency and stronger links to control-theoretic structure [4]. In addition, unified viewpoints connecting RL with dynamic programming and model predictive control help motivate control-oriented RL formulations and performance indices for real-time control [5]. Meanwhile, continuing progress in algorithmic design and discovery has expanded the practical deployment space of RL in complex systems [6], and complementary advances in scalable learning architectures and training heuristics further broaden applicability in robotics settings [7].

Despite these developments, achieving high-precision trajectory tracking for robotic manipulators using RL remains challenging in the presence of strong nonlinear coupling, parameter variations, friction, payload changes, and time-varying disturbances. Safety and constraint satisfaction motivate safe-RL mechanisms such as control barrier functions (CBFs) for enforcing state/input constraints [8]. Learning-based CBF formulations and DOB-assisted CBF designs further provide practical routes for integrating learning with safety guarantees under uncertainty [9].

In parallel, disturbance observers (DOBs) provide a computationally efficient means to estimate lumped uncertainties and reject matched disturbances in nonlinear systems [10]. DOB-based robust tracking control has been widely studied for manipulators under modeling errors and external disturbances [11]. Additional DOB/observer-enhanced robust control designs demonstrate improved tracking performance and robustness under practical disturbance conditions [12].

These considerations have motivated observer-integrated adaptive optimal control and reinforcement learning frameworks for uncertain nonlinear systems [13]. DOB-based actor–critic learning is one representative direction that combines online policy improvement with disturbance compensation [14]. Other DOB-enhanced ADP/RL schemes emphasize robustness and practical stability properties while learning in continuous time [15]. Disturbance-aware objective shaping has also been explored to better reflect uncertainty in the performance index used for learning and optimization [16]. Further, stability-oriented analyses are commonly developed to establish boundedness/UUB-type guarantees under approximation and observer assumptions [17]. Observer-integrated adaptive optimal control for robotic manipulators further extends these ideas toward real robotic platforms under disturbances and uncertainties [18].

However, in several DOB–RL implementations, the DOB is primarily designed and tuned independently, and its estimate is used mainly for feedforward compensation, while the learning objective and policy updates are only indirectly influenced by observer mismatch. Under time-varying disturbances, this separation may require the learning component to accommodate disturbance-induced transients and fast components, which can reduce learning efficiency and adversely affect control smoothness. These observations motivate a co-designed disturbance-aware actor–critic framework in which DOB information is embedded into the learning loop at both the state and objective levels, while preserving a control-theoretic structure suitable for real-time implementation.

The main contributions of this paper are summarized as follows.

1 **Disturbance-aware objective shaping:** A disturbance-aware performance index is formulated by explicitly incorporating the measurable DOB filter residual as a penalty term into the RL cost function. Unlike prior works [13,16–18] that construct the learning objective solely from tracking error and control effort — leaving the learning loop without a direct signal when the DOB estimate is inaccurate the proposed cost explicitly penalizes estimation mismatch, incentivizing the policy to operate in regions where the observer performs well.
2 **Co-designed DOB–Actor–Critic learning rule:** The actor update law is augmented with a disturbance-aware gradient term derived from the measurable residual, and the learning state includes both the disturbance estimate and the filter residual. This addresses two gaps absent from [13,14,17,18]: (i) cascade designs provide no gradient signal preventing the policy from selecting torques that amplify the DOB residual; and (ii) augmenting the state with the disturbance estimate alone cannot distinguish a well-converged observer from a poorly converged one, whereas including the filter residual allows the critic to condition value estimation on current observer quality.
3 **Composite control architecture for manipulators:** A composite torque law integrates a nominal/robust baseline, DOB compensation, and an actor corrective term, consistent with [10–12,19,20]. Unlike cascade architectures where the DOB and AC blocks operate independently, the proposed structure couples the DOB output into the learning loop at both the state level and the objective level, ensuring the actor corrective term is learned with explicit awareness of current observer performance rather than treating the DOB-compensated plant as a fixed, uncertainty-free system.
4 **Stability-oriented analysis:** A Lyapunov-based analysis is provided to establish boundedness (UUB-type) of closed-loop signals under standard observer and function-approximation assumptions, aligning online learning with stability requirements in continuous-time control [13–18].
5 **Progressive evaluation and scalability:** The method is evaluated on a 2-DOF manipulator and further assessed on a 6-DOF industrial manipulator under composite disturbances, with comparisons to DOB-only, standard actor–critic, and DOB-based ADP/RL baselines [13–18]. Fault-tolerant RL control of manipulators under actuator faults further motivates evaluation under non-ideal actuator conditions [21]. Modern dynamics and simulation/control references are also included to support modeling and implementation practices [22–24].

## 2. Related work

This section provides a brief review of the foundational research informing the present study. While RL for robotic control has been extensively studied for optimal control [1]– [6], achieving robust, real-time deployment under modeling errors and disturbances remains a persistent challenge [7]. To ensure reliability, safety and constraint enforcement through Control Barrier Functions (CBFs) has emerged as a key area of interest, particularly when integrated with RL and disturbance-aware filtering [8,9]. Complementary to this, DOB for robust tracking offers an efficient means of estimating lumped uncertainties in nonlinear manipulators, though its efficacy is often limited by bandwidth-noise trade-offs [10]– [12].

The key technical distinction of the proposed framework is threefold. First, prior DOB-RL designs ([13,16–18]) formulate the RL objective independently of DOB estimation quality: the cost function penalizes tracking error and control effort but not disturbance mismatch. In DACRL, the measurable filter residual $\tilde{𝐫}=𝐫-\hat{𝐝}$ appears directly in the cost via a $\lambda |\tilde{𝐫}{|}^{\text{2}}$ penalty, creating an explicit learning incentive to reduce estimation mismatch. Second, the actor update in DACRL includes a gradient term $\Delta_{W_{a}}|\tilde{𝐫}{|}^{\text{2}}$, which discourages policies that amplify the DOB residual a mechanism absent from all cascade designs reviewed. Third, both $\hat{𝐝}$ and $\tilde{𝐫}$ are included in the learning state, allowing the critic to condition value estimation on current observer quality rather than just the estimated disturbance magnitude.

Table 1 summarizes representative lines of work and highlights that this paper explicitly couples DOB mismatch into the learning objective and actor update, and evaluates the method on both 2-DOF and 6-DOF manipulators under modern control metrics.

**Table 1:** Representative DOB and learning-based control works summarized by design axes.

Ref.	Scope	DOB	Learning	DOB–learning coupling (objective/update)	Constraints/ safety	Fault tolerance	Stability analysis (reported)	Manipulator evaluation
[13]	Uncertain nonlinear systems	Yes	Yes (AC)	Yes	No	No	Boundedness/ stability-oriented	Not specified
[14]	Continuous-time nonlinear systems	No (disturbances modeled)	Yes (AC)	—	No	No	Robust/ stability-oriented	Not specified
[15]	Nonlinear systems	Observer (not DOB)	Yes (ADP/RL)	—	No	No	Stability-oriented	Not specified
[16]	Nonlinear systems	Yes (compensation)	Yes (RL)	Partial	No	No	Robust (H-infinity-motivated)	Not specified
[17]	Nonlinear systems → manipulators	Yes	Yes (RL)	Partial	No	No	Reported (varies by setting)	Yes (DOF not stated)
[18]	Robotic manipulators	Yes	Yes (ADP/RL)	Yes	No	No	Reported (robust/optimal)	Yes (DOF not stated)
[19]	Robot manipulators	Yes	NN (not RL)	—	No	No	Robust/ smooth	Yes (DOF not stated)
[20]	Robot manipulators	Yes	Adaptive fuzzy (not RL)	—	Yes (input saturation)	No	Finite-time	Yes (DOF not stated)
[21]	Robot manipulators	No	Yes (RL)	—	No	Yes (actuator faults)	Finite-time	Yes (DOF not stated)
**This work**	**2-DOF → 6-DOF (RV-2FBD)**	**Yes**	**Yes (AC)**	**Yes (objective shaping + co-designed update)**	**No (future: CBF)**	**No**	**Lyapunov/ UUB-type**	**Yes (2-DOF + 6-DOF)**

## 3. Formulation and analysis

### 3.1. Dynamics, tracking objective, and lumped disturbance model

Consider an (n)-DOF rigid manipulator described by the Euler–Lagrange dynamics:


$$\begin{array}{c} 𝐌\left(𝐪\right)\mathrm{\backslash stackrel}..𝐪+𝐂\left(𝐪,\mathrm{\backslash stackrel}.𝐪\right)\mathrm{\backslash stackrel}.𝐪+𝐠\left(𝐪\right)=\tau +𝐝\left(t\right) \end{array}$$ (1)

where $𝐪\in {\mathbb{R}}^{n}$ is the joint position, $\tau \in {\mathbb{R}}^{n}$ is the commanded torque, and **d**(t) denotes a matched lumped disturbance aggregating friction, external perturbations, and modeling errors. Such modeling is standard in multibody and robot dynamics and industrial robot simulation studies [22].

Given a desired trajectory ${𝐪}_{d}\left(t\right)$ define the tracking errors:


$$\begin{array}{c} 𝐞=𝐪-{𝐪}_{d},\text{\textbackslash{}hspace\{1em}\;\}\mathrm{\backslash stackrel}.𝐞=\mathrm{\backslash stackrel}.𝐪-{\mathrm{\backslash stackrel}.𝐪}_{d} \end{array}$$ (2)

and the filtered and sliding tracking variable:


$$\begin{array}{c} 𝐬=\mathrm{\backslash stackrel}.𝐞+\Lambda 𝐞,\text{\textbackslash{}hspace\{1em}\;\}\Lambda ≻0. \end{array}$$ (3)

***Assumption A1.*** The lumped disturbance **d**(t) is bounded and piecewise differentiable: $\left|𝐝\left(t\right)\right|\leq \overset{―}{d}$ as commonly assumed in disturbance-observer-based robust control [10].

### 3.2. Measurable residual and disturbance observer smoothing

Because **d**(t) is not measurable, we define a measurable residual computed from the nominal model and measured signals:


$$\begin{array}{c} 𝐫\left(t\right)=𝐑\left(𝐪\left(t\right),\dot{𝐪}\left(t\right),\ddot{𝐪}\left(t\right),\tau \left(t\right)\right) \end{array}$$ (4)

Residual and DOB constructions are widely used for robot disturbance estimation and robust tracking [11]. In practice, the residual contains modeling and sensing errors.


$$\begin{array}{c} 𝐫\left(t\right)=𝐝\left(t\right)+\varepsilon \left(t\right) \end{array}$$ (5)

where ε(t) is a bounded lumped error term, $\left|\varepsilon \left(t\right)\right|\leq \overset{―}{\varepsilon }$.

To obtain a smooth disturbance estimate, we employ a first-order DOB filter:


$$\begin{array}{c} \dot{\hat{𝐝}}\left(t\right)=\ell \left(𝐫\left(t\right)-\hat{𝐝}\left(t\right)\right),\;\;\ell >0 \end{array}$$ (6)

Define the (unmeasurable) true estimation error and the measurable filter residual:


$$\begin{array}{c} \tilde{𝐝}\left(t\right)=𝐝\left(t\right)-\hat{𝐝}\left(t\right),\text{\textbackslash{}hspace\{1em}\;\}\tilde{𝐫}\left(t\right)=𝐫\left(t\right)-\hat{𝐝}\left(t\right) \end{array}$$ (7)

Combining (5) and (7) yields:


$$\begin{array}{c} \tilde{r}\left(t\right)=\tilde{d}\left(t\right)+\varepsilon \left(t\right) \end{array}$$ (8)

and hence the two-sided bound:


$$\begin{array}{c} \left|\tilde{𝐝}\left(t\right)\right|\leq \left|\tilde{𝐫}\left(t\right)\right|+\left|\varepsilon \left(t\right)\right|,\text{\textbackslash{}hspace\{1em}\;\}\left|\tilde{𝐫}\left(t\right)\right|\leq \left|\tilde{𝐝}\left(t\right)\right|+\left|\varepsilon \left(t\right)\right| \end{array}$$ (9)

Since **d(**t) is not measurable, directly minimizing $|𝐝-\hat{𝐝}{|}^{\text{2}}=|\tilde{𝐝}{|}^{\text{2}}$ is not implementable online. A principled measurable surrogate is instead: $|\tilde{𝐫}(t){|}^{\text{2}}=|𝐫\left(t\right)-\hat{𝐝}\left(t\right){|}^{\text{2}}$ because (9) shows that reducing $\left|\tilde{𝐫}\right|$ reduces $\left|\tilde{𝐝}\right|$ up to the bounded modeling/sensing term |ε|. Penalizing $|𝐫{|}^{\text{2}}$ is weaker because it mixes disturbance magnitude with ε; therefore, this work uses $|\tilde{𝐫}{|}^{\text{2}}$ as the disturbance-aware mismatch penalty, consistent with DOB-based RL co-design motivations in the recent literature [13].

### 3.3. Disturbance-aware RL objective, learning state, and analysis-compatible parameterization

We form a disturbance-aware learning state:


$$\begin{array}{c} 𝐱={\left[\begin{array}{ccccc} @ccccc@{𝐞}^{⊤} & {\dot{𝐞}}^{⊤} & {𝐬}^{⊤} & {\hat{𝐝}}^{⊤} & {\tilde{𝐫}}^{⊤} \end{array}\right]}^{⊤} \end{array}$$ (10)

Define the instantaneous utility:


$$\begin{array}{c} 𝐔\left(𝐱,\tau \right)={𝐞}^{⊤}𝐐𝐞+\tau^{⊤}R\tau +\lambda |\tilde{𝐫}{|}^{\text{2}}\text{\textbackslash{}hspace\{1em}\}𝐐≽0,𝐑≻0,\lambda \geq 0 \end{array}$$ (11)

which combines tracking accuracy, control effort, and the measurable disturbance mismatch penalty.

The $\lambda |\tilde{𝐫}{|}^{\text{2}}$ penalty in 𝐔(𝐱,τ) constitutes the primary departure from standard actor-critic objectives. Whereas conventional RL objectives for manipulators penalize tracking error and control effort [1,2,14], the inclusion of the measurable residual energy creates a direct coupling between observer performance and the learning signal. This incentivizes the policy to operate in regions where the DOB provides accurate estimates, rather than relying solely on the nominal+robust baseline to absorb remaining estimation error.

In discrete time, the discounted value function is:


$$\begin{array}{c} 𝐕\left({𝐱}_{k}\right)=\sum_{j=0}^{\infty }\gamma^{j}𝐔\left({𝐱}_{k+j},\tau_{k+j}\right),\text{\textbackslash{}hspace\{1em}\;\}0<\gamma <1 \end{array}$$ (12)

This objective is consistent with optimal-control and RL formulations grounded in dynamic programming and modern RL surveys [3].

To ensure strict consistency between stability-oriented analysis and simulation and implementation, both actor and critic use linear-in-weights approximators

**Actor (policy):**


$$\begin{array}{c} {𝐮}_{a}\left(x\right)={𝐖}_{a}^{⊤}\phi \left(x\right) \end{array}$$ (13)

**Critic (value):**


$$\begin{array}{c} \hat{𝐕}\left(x\right)={𝐖}_{c}^{⊤}\psi \left(x\right) \end{array}$$ (14)

where $\phi \left(x\right)\in {\mathbb{R}}^{N_{a}}$ is a bounded feature and basis vector and $\psi \left(x\right)\in {\mathbb{R}}^{N_{c}}$ is an RBF feature vector standard in disturbance-aware actor–critic and robust RL control for nonlinear systems [13].


$$\begin{array}{c} \phi \left(x\right)=\text{sat}\left(\left[\begin{array}{c} @c@\begin{array}{c} 1\text{\textbackslash{}hspace\{0.33em}\}\\ 𝐞\text{\textbackslash{}hspace\{0.33em}\}\\ \dot{𝐞}\\ \text{\textbackslash{}hspace\{0.33em}\}𝐬\text{\textbackslash{}hspace\{0.33em}\}\\ \hat{𝐝}\\ \text{\textbackslash{}hspace\{0.33em}\}\tilde{𝐫}\text{\textbackslash{}hspace\{0.33em}\}\\ 𝐞⊙𝐞\text{\textbackslash{}hspace\{0.33em}\}\\ 𝐬⊙𝐬 \end{array} \end{array}\right]\right),\text{\textbackslash{}hspace\{1em}\;\}\psi_{i}\left(x\right)=exp\left(-\frac{|x-c_{i}{|}^{\text{2}}}{\sigma_{i}^{\text{2}}}\right) \end{array}$$ (15)

where sat(·) is component-wise saturation and normalization and $c_{i},\sigma_{i}$ are the RBF centers and widths.

***Assumption A2.*** On a compact set 𝒳 there exist ideal weights ${𝐖}_{c}^{*},\text{\textbackslash{}hspace\{0.17em}\}{𝐖}_{a}^{*}$ such that:


$$\begin{array}{c} 𝐕\left(x\right)={𝐖}_{c}^{*⊤}\psi \left(x\right)+\varepsilon_{V}\left(x\right),\text{\textbackslash{}hspace\{1em}\}u^{\left(x\right)}={𝐖}_{a}^{*⊤}\phi \left(x\right)+\varepsilon_{u}\left(x\right),\text{\textbackslash{}hspace\{1em}\}\left|\varepsilon_{V}\right|\leq {\overset{―}{\varepsilon }}_{V},\text{\textbackslash{}hspace\{0.17em}\}\left|\varepsilon_{u}\right|\leq {\overset{―}{\varepsilon }}_{u} \end{array}$$ (16)

This assumption and the bounded-weight mechanism below are consistent with robust actor–critic learning under matched disturbances [14].

### 3.4. Unified TD error and implementable update laws

Define the temporal-difference (TD) error:


$$\begin{array}{c} \delta_{k}=𝐔\left({𝐱}_{k},\tau_{k}\right)+\gamma \hat{𝐕}\left({𝐱}_{k+1}\right)-\hat{𝐕}\left({𝐱}_{k}\right) \end{array}$$ (17)

To guarantee bounded weights and align with UUB-style reasoning, use gradient descent with projection onto compact sets $\Omega_{a},\Omega_{c}$.

**Critic update:**


$$\begin{array}{c} {𝐖}_{c,k+1}=\Pi_{\Omega_{c}}\left({𝐖}_{c,k}-\eta_{c}\delta_{k}\psi \left(x_{k}\right)\right) \end{array}$$ (18)

**Actor update:**


$$\begin{array}{c} {𝐖}_{a,k+1}=\Pi_{\Omega_{a}}({𝐖}_{a,k}-\eta_{a}g_{k}^{\text{PI}}+\lambda g_{k}^{\text{DA}}) \end{array}$$ (19)

where $g^{\text{PI}}k$ denotes a standard actor–critic policy-improvement surrogate used in robust RL control and the disturbance-aware term is defined through the measurable mismatch:


$$\begin{array}{c} g^{\text{DA}}k=\nabla {𝐖}_{a}|{\tilde{𝐫}}_{k}{|}^{\text{2}},\text{\textbackslash{}hspace\{0.17em}\;\}{\tilde{𝐫}}_{k}={𝐫}_{k}-{\hat{𝐝}}_{k},\text{\textbackslash{}hspace\{0.17em}\;\}\nabla {𝐖}_{a}u_{a}(x_{k})=\phi (x_{k}) \end{array}$$ (20)

The disturbance-aware actor gradient term $g^{\text{DA}}k=\nabla {𝐖}_{a}|{\tilde{𝐫}}_{k}{|}^{\text{2}}$ represents a second departure from standard policy improvement. In conventional actor-critic updates, the policy is updated to reduce the TD error, which reflects cumulative discounted cost. The additional gradient $\nabla {𝐖}_{a}|{\tilde{𝐫}}_{k}{|}^{\text{2}}$ provides a direct regularization signal that penalizes policies generating large DOB residuals, independent of the TD error. This discourages the actor from selecting torques that, while effective in reducing tracking error, destabilize DOB estimation or amplify unmodeled dynamics.

In the proposed DACRL framework, the DOB has a dual role. In the control loop, the disturbance estimate $\hat{𝐝}$ is used directly in the composite torque law to compensate for matched lumped disturbances. In the learning loop, $\hat{𝐝}$ and the measurable DOB mismatch and residual are incorporated into the disturbance-aware state and objective function, so that the actor–critic updates are influenced by disturbance-estimation quality. Beyond mere compensation, the DOB’s disturbance estimates directly shape both control torque and policy adaptation. For clarity and reproducibility, the online implementation of the proposed DACRL controller is summarized in Algorithm 1. At each sampling instant, the controller first computes the tracking errors and the measurable disturbance residual. The DOB’s disturbance estimate provides feedforward compensation and builds an augmented state, driving the actor’s control output and the critic’s evaluation. The composite torque is applied to the manipulator, and the actor–critic weights are updated using the TD error with projection to maintain bounded adaptation.

**Algorithm 1.**

Inputs: ${𝐪}_{k},{\dot{𝐪}}_{k},{𝐪}_{d,k},{\dot{𝐪}}_{d,k},{\ddot{𝐪}}_{d,k}$, parameters $𝐐,𝐑,\Lambda ,\ell ,\gamma ,\eta_{a},\eta_{c},\lambda$ and bounds $\Omega_{a},\Omega_{c}$.

At each sampling instant k:

1. Compute ${𝐞}_{k},{\dot{𝐞}}_{k}$ via (2), and ${𝐬}_{k}$ via (3).

2. Compute residual ${𝐫}_{k}$ via (4).

3. Update DOB estimate (Euler discretization of (6)

${\hat{𝐝}}_{k+1}={\hat{𝐝}}_{k}+T_{s}\ell ({𝐫}_{k}-{\hat{𝐝}}_{k})$ compute ${\tilde{𝐫}}_{k}={𝐫}_{k}-{\hat{𝐝}}_{k}$ via (7).

4. Form learning state $x_{k}$ via (10).

5. Compute features $\phi \left(x_{k}\right)$ and $\psi \left(x_{k}\right)$ via (15).

6. Evaluate critic: $\hat{𝐕}\left(x_{k}\right)={𝐖}_{c,k}^{⊤}\psi \left(x_{k}\right)$ via (14).

7. Synthesize composite control torque:

$\tau_{k}=\tau_{\text{nom}}+\tau_{\text{rob}}+\tau_{\text{DOB}}+{𝐮}_{a}\left(x_{k}\right)\text{\textbackslash{}hspace\{0.17em}\}with\text{\textbackslash{}hspace\{0.17em}\}{𝐮}_{a}$ from (13).

8. Compute utility $U_{k}$ via (11).

9. After observing $x_{k+1}$ compute TD error $\delta_{k}$ via (17).

10. Update $W_{c}$ and $W_{a}$ via (18) – (20) with projection and clipping.

Algorithm 1 highlights that the proposed method is implemented fully online. The DOB, actor, critic, and composite torque law are updated at every sampling instant using measurable signals. The projection operation in the actor–critic updates is included to prevent parameter drift and to maintain consistency with the boundedness assumptions used in the stability analysis.

## 4. Controller design

This section provides a reproducible design recipe for the proposed DACRL controller. It presents the manipulator model and assumptions, the DOB and DACRL components, and the final composite torque law for real-time implementation on 2-DOF and higher-DOF platforms. The 2-DOF manipulator is specified in Table 2 and illustrated in Fig 1.

**Table 2:** Robot manipulator parameters.

Parameter	Symbol	Value	Unit
Inertia Coefficient 1	*p*_*1*_	3.473	kg ⋅ m^2^
Inertia Coefficient 2	*p*_*2*_	0.196	kg ⋅ m^2^
Inertia Coefficient 3	*p*_*3*_	0.242	kg ⋅ m^2^
Joint 1 Friction	*f*_*d1*_	5.3	N ⋅ m ⋅ s
Joint 2 Friction	*f*_*d2*_	1.1	N ⋅ m ⋅ s
Link Length 1	*l_1_*	0.5	m
Link Length 2	*l_2_*	0.5	m

**Fig 1:**
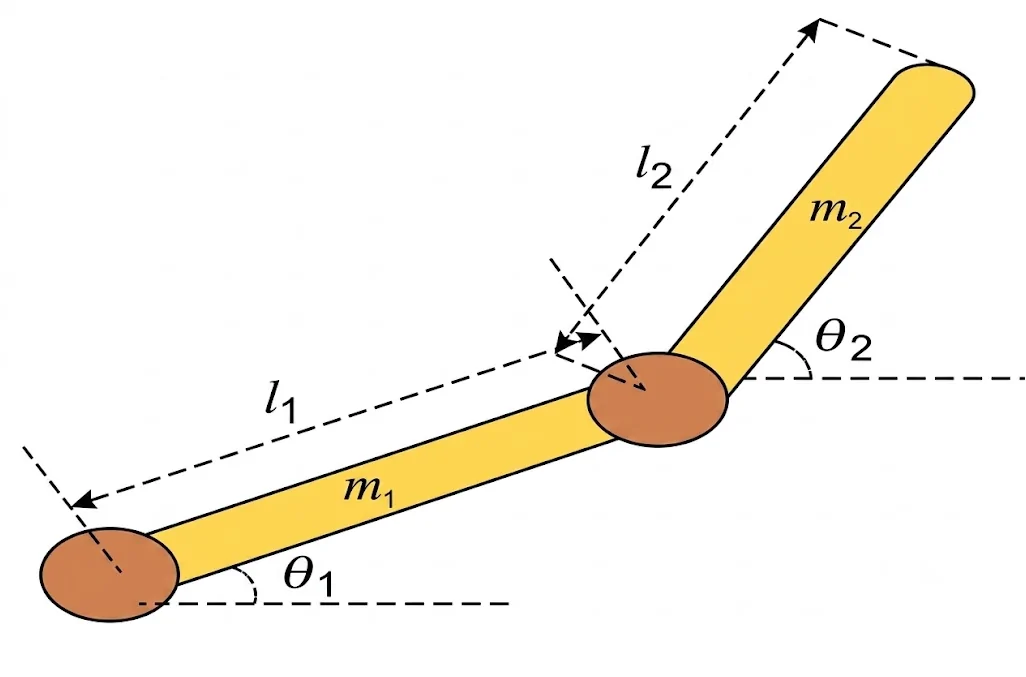
Mechanical model of the 2-DOF robot.

Consider an (n)-DOF rigid-link manipulator with joint coordinates $𝐪\in {\mathbb{R}}^{n}$, joint velocity $\dot{𝐪}\in {\mathbb{R}}^{n}$ and input torque $\tau \in {\mathbb{R}}^{n}$. The dynamics can be written in Euler–Lagrange form as:


$$\begin{array}{c} 𝐌(𝐪)\ddot{𝐪}+𝐂(𝐪,\dot{𝐪})\dot{𝐪}+𝐠(𝐪)=𝐁(𝐪)\tau +𝐝(𝐭) \end{array}$$ (21)

where $𝐌(𝐪)\in {\mathbb{R}}^{n\times n}$ is symmetric positive definite, $𝐂(𝐪,\dot{𝐪})\dot{𝐪}\in {\mathbb{R}}^{n}$ collects Coriolis/centrifugal terms, $𝐠\left(𝐪\right)\in {\mathbb{R}}^{n}$ is the gravity vector, $𝐁\left(𝐪\right)\in {\mathbb{R}}^{n\times n}$ is the input-channel mapping, and $𝐝\left(t\right)\in {\mathbb{R}}^{n}$ denotes lumped matched disturbances.

Assumption 1. 𝐁(𝐪) is nonsingular for all **q** in the operating region, which ensures disturbances entering through the same channel as control can be compensated. For standard torque-controlled manipulators, $𝐁\left(𝐪\right)=I_{n}$.

The lumped disturbance aggregates multiple non-idealities:


$$\begin{array}{c} 𝐝\left(t\right)={𝐝}_{\text{ext}}\left(t\right)+{𝐝}_{\text{fric}}\left(𝐪,\dot{𝐪}\right)+{𝐝}_{\text{unm}}\left(𝐪,\dot{𝐪},\ddot{𝐪}\right) \end{array}$$ (22)

where ${𝐝}_{\text{ext}}\left(t\right)$ denotes external disturbances, ${𝐝}_{\text{fric}}\left(𝐪,\dot{𝐪}\right)$ includes viscous and Coulomb friction, and ${𝐝}_{\text{unm}}\left(𝐪,\dot{𝐪},\ddot{𝐪}\right)$ denotes unmodeled dynamics and parametric mismatch. In the proposed design, all terms in (22) are treated as a single matched disturbance entering the input channel of (21).

Given a desired trajectory ${𝐪}_{d}\left(t\right)$ define tracking errors:


$$\begin{array}{c} 𝐞≜𝐪-{𝐪}_{d},\text{\textbackslash{}hspace\{1em}\;\}\dot{𝐞}≜\dot{𝐪}-{\dot{𝐪}}_{d} \end{array}$$ (23)

and the sliding-like variable $𝐬\in {\mathbb{R}}^{n}$ used for baseline robustness:

$𝐬≜\dot{𝐞}+\Lambda 𝐞$ where $\Lambda =\text{diag}(\lambda_{\text{1}},\ldots ,\lambda_{n})≻0$.

A residual signal that is measurable from model terms and sensor signals is defined as


$$\begin{array}{c} 𝐫=𝐌(𝐪)\ddot{𝐪}+𝐂(𝐪,\dot{𝐪})\dot{𝐪}+𝐠(𝐪)-𝐁(𝐪)\tau \end{array}$$ (24)

If the model were exact and noise-free, then 𝐫=d(t). In practice**, r** also contains modeling error and measurement noise; therefore, we obtain a smoothed disturbance estimate via a first-order low-pass filter:


$$\begin{array}{c} \dot{\hat{𝐝}}=-\alpha \hat{𝐝}+\alpha 𝐫\text{\textbackslash{}hspace\{1em}\;\}\alpha >0 \end{array}$$ (25)

applied component-wise to each joint. The estimate $\hat{𝐝}\in {\mathbb{R}}^{n}$ is used (i) for disturbance compensation in the torque input, and (ii) as an additional disturbance context signal for learning.

Implementation note (acceleration availability). If $\ddot{𝐪}$ is not directly measured, it can be obtained from a filtered differentiator $\dot{\hat{\dot{𝐪}}}=\beta (\dot{𝐪}-\hat{\dot{𝐪}}),\text{\textbackslash{}hspace\{1em}\}\hat{\ddot{𝐪}}=\beta (\dot{𝐪}-\hat{\dot{𝐪}})$ with β>0 chosen to balance noise amplification. In that case, α should be tuned conservatively to avoid injecting differentiation noise into $\hat{𝐝}$. Larger α yields faster disturbance tracking but higher sensitivity to noise; smaller α yields smoother $\hat{𝐝}$ and torque but slower rejection.

To embed DOB information into the learning loop, we define the disturbance-aware state:


$$\begin{array}{c} 𝐱≜{\left[\begin{array}{cccc} @cccc@{𝐞}^{⊤} & {\dot{𝐞}}^{⊤} & {𝐬}^{⊤} & {\hat{𝐝}}^{⊤} \end{array}\right]}^{⊤}\in {\mathbb{R}}^{4n} \end{array}$$ (26)

This augmentation enables the policy and value function to explicitly condition on disturbance estimates and tracking “robustness” signals (s), supporting co-design rather than cascade compensation.

To maintain consistency with Lyapunov-style boundedness analysis, we employ linear-in-weights function approximation.

Actor (policy) approximation.


$$\begin{array}{c} {𝐮}_{a}\left(x\right)≜\hat{\pi }\left(x\right)={𝐖}_{a}^{⊤}\phi_{a}\left(x\right)\in {\mathbb{R}}^{n} \end{array}$$ (27)

where ${𝐖}_{a}\in {\mathbb{R}}^{N_{a}\times n}$ is the actor weight matrix, and $\phi_{a}\left(x\right)\in {\mathbb{R}}^{N_{a}}$ is a chosen basis vector as low-order polynomials and RBFs of $𝐞,\dot{𝐞},𝐬,𝐝$. This form is linear in ${𝐖}_{a}$ which is standard in stability-oriented adaptive and actor–critic control. Critic approximation:


$$\begin{array}{c} \hat{𝐕}\left(x\right)={𝐖}_{c}^{⊤}\phi_{c}\left(x\right)\in \mathbb{R} \end{array}$$ (28)

where ${𝐖}_{c}\in {\mathbb{R}}^{N_{c}}$ and $\phi_{c}\left(x\right)\in {\mathbb{R}}^{N_{c}}$ is typically an RBF basis. Define an instantaneous utility that reflects tracking and control effort objectives:


$$\begin{array}{c} \ell \left(𝐱,\tau \right)={𝐬}^{⊤}𝐐𝐬+\tau^{⊤}𝐑\tau ,\text{\textbackslash{}hspace\{1em}\;\}𝐐≽0,𝐑≻0 \end{array}$$ (29)

Using a discrete-time implementation with sampling $T_{s}$ is defined the TD error:


$$\begin{array}{c} \delta_{k}=\ell \left({𝐱}_{k},\tau_{k}\right)+\gamma \hat{𝐕}\left({𝐱}_{k+1}\right)-\hat{𝐕}\left({𝐱}_{k}\right),\text{\textbackslash{}hspace\{1em}\;\}\gamma \in \left(0,1\right) \end{array}$$ (30)

Critic update gradient descent with projection:


$$\begin{array}{c} {𝐖}_{c,k+1}=\Pi_{\Omega_{c}}\left({𝐖}_{c,k}-\eta_{c}\delta_{k}\nabla_{W_{c}}\hat{𝐕}\left(x_{k}\right)\right)=\Pi_{\Omega_{c}}\left({𝐖}_{c,k}-\eta_{c}\delta_{k}\phi_{c}\left(x_{k}\right)\right) \end{array}$$ (31)

where $\eta_{c}>0$ is the critic learning rate and $\Pi_{\Omega_{c}}(\cdot )$ denotes projection onto a compact set $\Omega_{c}$ to prevent parameter drift.

Actor update (disturbance-aware co-design). We design the actor update to include (i) a standard policy-improvement term, and (ii) a measurable coupling penalty driven by DOB mismatch. A practical measurable surrogate is the squared residual energy ${𝐉}_{d}=|{𝐫}_{k}{|}^{\text{2}}$, since ${𝐫}_{k}$ is measurable from (24). The actor update is:


$$\begin{array}{c} {𝐖}_{a,k+1}=\Pi_{\Omega_{a}}\left({𝐖}_{a,k}-\eta_{a}(\underset{\text{policy-improvement}}{\underbrace{\nabla_{W_{a}}{𝐉}_{\pi }}}+\lambda_{d}\underset{\text{DOB-learning coupling}}{\underbrace{\nabla {𝐖}_{a}|{𝐫}_{k}{|}^{\text{2}}}})\right) \end{array}$$ (32)

where $\eta_{a}>0$ is the actor learning rate and $\lambda_{d}\geq 0$ weights the coupling.

A concrete choice consistent with measurability is: Use $J_{\pi }=\hat{V}\left(x_{k}\right)$ giving; For the coupling term, from (24) we have ${𝐫}_{k}=-𝐁\left({𝐪}_{k}\right)\tau_{k}$. Since $\tau_{k}$ contains the actor term, $\nabla_{W_{a}}|{𝐫}_{k}{|}^{\text{2}}$ can be computed as:

$\nabla_{W_{a}}|{𝐫}_{k}{|}^{\text{2}}=2{𝐫}_{k}^{⊤}\nabla_{W_{a}}{𝐫}_{k}=-2{𝐫}_{k}^{⊤}𝐁({𝐪}_{k})\nabla_{W_{a}}\tau_{k}$, which is measurable because ${𝐫}_{k},\text{\textbackslash{}hspace\{0.17em}\;\}𝐁\left({𝐪}_{k}\right)$ and $\nabla_{W_{a}}\tau_{k}$ are known online. For linear actor (27), $\nabla_{W_{a}}\tau_{k}$ is directly related to $\phi_{a}\left(x_{k}\right)$.

The final control input is a composite torque:


$$\begin{array}{c} \tau =\tau_{\text{nom}}+\tau_{\text{rob}}+\tau_{a}-𝐁(q{)}^{-1}\hat{𝐝} \end{array}$$ (33)

where $\tau_{a}≜{𝐮}_{a}\left(x\right)={𝐖}_{a}^{⊤}\phi_{a}\left(x\right)$ is the actor term.

Nominal (computed-torque-like) term.


$$\begin{array}{c} \tau_{\text{nom}}=𝐁(𝐪{)}^{-1}\left(𝐌\left(𝐪\right){\ddot{𝐪}}_{r}+𝐂\left(𝐪,\dot{𝐪}\right){\dot{𝐪}}_{r}+𝐠\left(𝐪\right)\right),\text{\textbackslash{}hspace\{1em}\;\}{\dot{𝐪}}_{r}≜{\dot{𝐪}}_{d}-\Lambda 𝐞,{\ddot{𝐪}}_{r}≜{\ddot{𝐪}}_{d}-\Lambda \dot{e} \end{array}$$ (34)

This term provides baseline model-based tracking.

Robust term (smooth sliding saturation).


$$\begin{array}{c} \tau_{\text{rob}}=-𝐁(q{)}^{-1}\left({𝐊}_{s}\text{sat}\left(\frac{𝐬}{\varepsilon }\right)\right)\text{\textbackslash{}hspace\{1em}\;\}{𝐊}_{s}=\text{diag}(k_{s,1},\ldots ,k_{s,n})≻0 \end{array}$$ (35)

where sat(·) is applied element-wise and ε>0 controls smoothness (larger ε → smoother torque, smaller ε → stronger sliding action but may induce chatter).

Remarks. $\tau_{\text{nom}}+\tau_{\text{rob}}$ ensures a stabilizing baseline and torque smoothness. $-{𝐁}^{-1}\hat{𝐝}$ offloads fast disturbance rejection to the DOB. $\tau_{a}$ improves performance online under the disturbance-aware state x, while the coupling in (32) discourages policies that increase residual mismatch.

The design in (21) – (35) is directly applicable to (n)-DOF manipulators by using vector, matrix forms with $𝐪,\dot{𝐪},\ddot{𝐪},\tau ,𝐝,\hat{𝐝}\in {\mathbb{R}}^{n}$. The primary scaling considerations are: (i) basis size $N_{a},N_{c}$ for $\phi_{a}$, $\phi_{c}$, (ii) DOB filter gain α_per_ joint or joint-dependent $\alpha_{i}$, and (iii) robust.

The engineering motivation for the disturbance-aware coupling term in the actor update is to prevent the policy from selecting torque commands that, while reducing tracking error in the short term, amplify uncompensated disturbance components and degrade observer convergence. A standard actor update driven solely by the TD error carries no such signal. By explicitly penalizing the DOB filter residual in the policy gradient, the actor is guided to operate the plant in regimes where the disturbance observer remains effective, thereby preserving the disturbance rejection capability that the composite control architecture relies upon.

## 5. Simulation scenarios and results

### 5.1. Simulation setup

All simulations were implemented in Python 3.10 using PyTorch 2.2 and SciPy 1.13 for numerical integration. The DACRL controller is implemented online with a fixed sampling time of Ts = 1 ms. The plant dynamics are integrated using a fourth-order Runge–Kutta method, while the actor, critic, and DOB are updated at every sampling instant. The actor adopts a linear-in-weights policy using normalized and clipped handcrafted polynomial/RBF features of the disturbance-aware state. The critic uses an RBF-based linear-in-weights value function. RBF centers are selected by k-means clustering from a short warm-up rollout, and RBF widths are chosen from the mean distance to neighboring centers. The actor and critic weights are updated by projected gradient descent, with projection bounds used to avoid parameter drift. Convergence is evaluated empirically through bounded actor/critic losses, bounded weights, reduced TD error, bounded DOB estimation error, post-transient tracking RMSE, peak tracking error, and torque-energy behavior. To maintain consistency with the Lyapunov/UUB-oriented analysis, both learning components follow the linear-in-weights approximation used in (27)–(32). This design prioritizes analytical tractability, bounded online adaptation, and real-time feasibility. Nevertheless, it may sacrifice approximation capacity compared with deep neural networks, and the impact of higher-capacity approximators will be investigated in future work. Specifically, the actor is parameterized as ${𝐮}_{a}\left(x\right)={𝐖}_{a}^{⊤}\phi \left(x\right)$ where ϕ(x) is a handcrafted basis vector. The critic is parameterized as $\hat{𝐕}\left(x\right)={𝐖}_{c}^{⊤}\psi \left(x\right)$ where ψ(x) is an RBF feature vector. Online updates use per-step gradient descent with projection (31)–(32) to keep weights bounded and avoid parameter drift, rather than deep-network backpropagation. RBF centers were obtained by k-means clustering over state samples collected from a short warm-up rollout; widths were set to the mean distance to the k-nearest centers. The nominal parameters in Tables 3 and 4 were selected to obtain stable online learning and smooth torque responses; however, the influence of these parameters on tracking accuracy, torque energy, and disturbance-estimation performance should be further quantified through a dedicated sensitivity study. Controller parameters were fixed as in Table 3. In all experiments, features were normalized and clipped to predefined ranges to satisfy boundedness assumptions in the analysis. The actor basis size was set to $N_{a}=\text{dim}\left(\phi \right)$ and the critic RBF size was set to $N_{c}=\text{dim}\left(\psi \right)$ as reported in Table 4. Projection was implemented via component-wise clipping $𝐖\leftarrow \text{clip}(𝐖,-{𝐖}_{max},{𝐖}_{max})$.

**Table 3:** Parameters controller.

Parameter	Value	Description
*k_1_*	30	Tracking gain 1
*k_2_*	10	Tracking gain 2
*k_n_*	10	Robustness gain
*α*	20	Disturbance observer gain
*γ*	0.1	Discount factor
***Q***	diag([50, 200])	State weighting matrix
***R***	diag([0.1, 0.1])	Control weighting matrix
λ	0.5	Disturbance penalty
$\alpha_{a},\alpha_{c}$	$10^{-4}$	Learning rate

**Table 4:** Benchmark reproducibility.

Parameter	Actor	Critic
Approximation type	Linear-in-weights $u_{a}\left(x\right)={𝐖}_{a}^{⊤}\phi \left(x\right)\left(27\right)$	RBF linear-in-weights $\hat{𝐕}\left(x\right)={𝐖}_{c}^{⊤}\psi \left(x\right)\left(28\right)$
Feature size	$N_{a}=\text{dim}\left(\phi \right)$ (reported per experiment)	$N_{c}=\text{dim}\left(\psi \right)$ (reported per experiment)
Features	ϕ(x): low-order polynomials and/or RBFs of disturbance-aware state x	ψ(x): RBF feature vector
Learning rate	$\eta_{a}=1\times 10^{-3}$	$\eta_{c}=5\times 10^{-4}$
Discount factor γ	—	0.1
Update rule	Gradient descent + projection (32)	Gradient descent + projection (31)
Projection and bounds	Component-wise clipping to compact set ${𝐖}_{a}|\leq {𝐖}_{a,max}$	Component-wise clipping to compact set $\left|{𝐖}_{c}\right|\leq {𝐖}_{c,max}$
Update frequency	Every step (1ms)	Every step (1ms)

For fair benchmarking and to isolate the contribution of the proposed co-design, all learning components in AC, OADP+DOB, and DACRL employed the same feature sizes and learning hyperparameters reported in Table 4.

Statistical robustness. Current results show DACRL’s effectiveness under fixed settings. To better quantify robustness and repeatability, future work will conduct multiple independent runs with varying conditions and report comprehensive statistical metrics.

### 5.2. Simulation results for scenario 1 with 2-DOF manipulator model

The proposed controller was tested on a realistic 2-DOF manipulator (Table 1) under complex time-varying disturbances composed of sinusoidal terms, periodic step changes, and amplitude variations to emulate payload shifts and persistent environmental interactions. The desired trajectory for both joints was set to a smooth, exponentially converging sinusoidal signal to avoid unrealistic initial jerks: $q_{d}\left(t\right)=A(1-e^{\left(-t\right)})sin\left(\varpi t\right)$ with A=[0.6, 0.8]^T^ rad/s and ω = π rad/s.

$q_{ref}\left(t\right)=0.6(1-e^{\left(-t\right)})sin\left(\pi t\right)$ (Joint 1)
$q_{ref}\left(t\right)=0.8(1-e^{\left(-t\right)})sin\left(\pi t\right)$ (Joint 2)

The trajectory tracking performance of the proposed DACRL controller is depicted in Fig 2. It is observed that both joint angles (*q*_*1*_, *q*_*2*_) accurately track their respective reference trajectories (*q*_*1d*_, *q*_*2d*_) after a brief initial transient phase.

**Fig 2:**
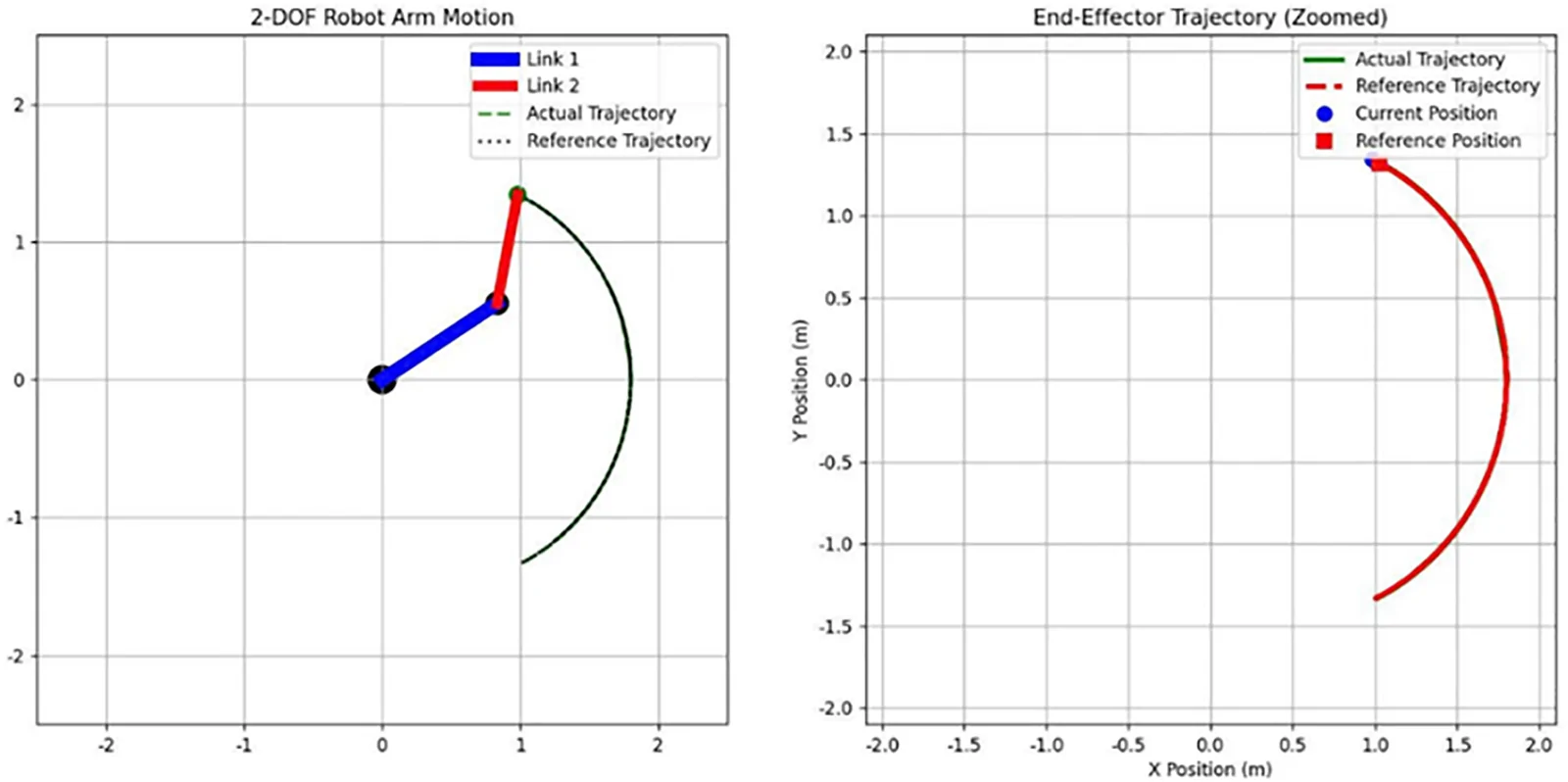
DOF robot arm motion and trajectory tracking in Workspace.

The left panel shows the arm configuration alongside the actual and reference end-effector trajectories, while the right panel provides a zoomed Cartesian view. Qualitatively, the actual trajectory closely follows the reference path with no visible steady-state offset, drift, or high-frequency chattering, confirming effective disturbance rejection by the composite controller. Quantitatively, the close Cartesian agreement is consistent with the joint-space steady-state errors of ±0.02 rad (Joint 1) and ±0.05 rad (Joint 2) reported in Fig 4, and the smooth trajectory curvature confirms that the DOB successfully suppresses the injected time-varying disturbances without inducing oscillations. From an engineering perspective, the results demonstrate that joint-level disturbance rejection translates directly into task-space tracking precision, which is the practically relevant performance criterion for manipulation applications.

Fig 3 demonstrates exceptional tracking performance, with both joints converging to their references within 0.5s. The small overshoot <5% in Joint 2 indicates well-tuned control gains.

**Fig 3:**
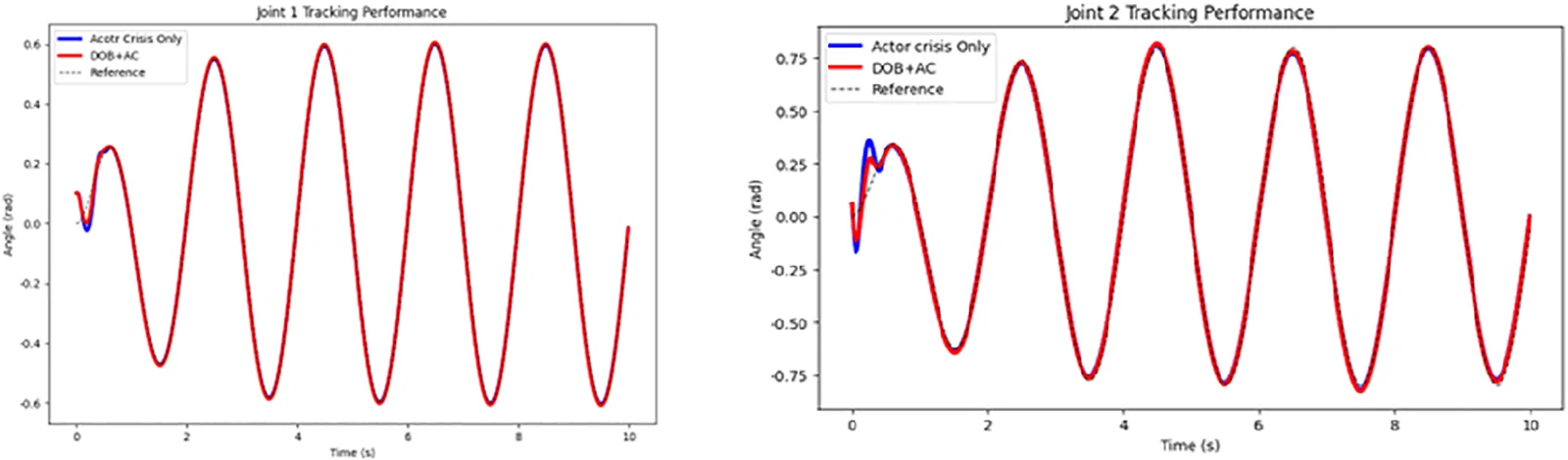
Joint trajectory tracking.

With the DOB + AC configuration exhibiting a noticeably smaller initial transient than the Actor-Crisis-Only baseline, and both methods achieving near-identical steady-state tracking thereafter, confirming that the DOB contribution is most significant during the transient phase.

The corresponding tracking errors are presented in Fig 4. The steady-state tracking errors are confined within ±0.02 rad for Joint 1 and ±0.05 rad for Joint 2, which represents a high degree of tracking precision. The controller successfully suppresses the effects of significant nonlinearities and the complex, injected external disturbances, confirming the theoretical UUB stability result.

**Fig 4:**
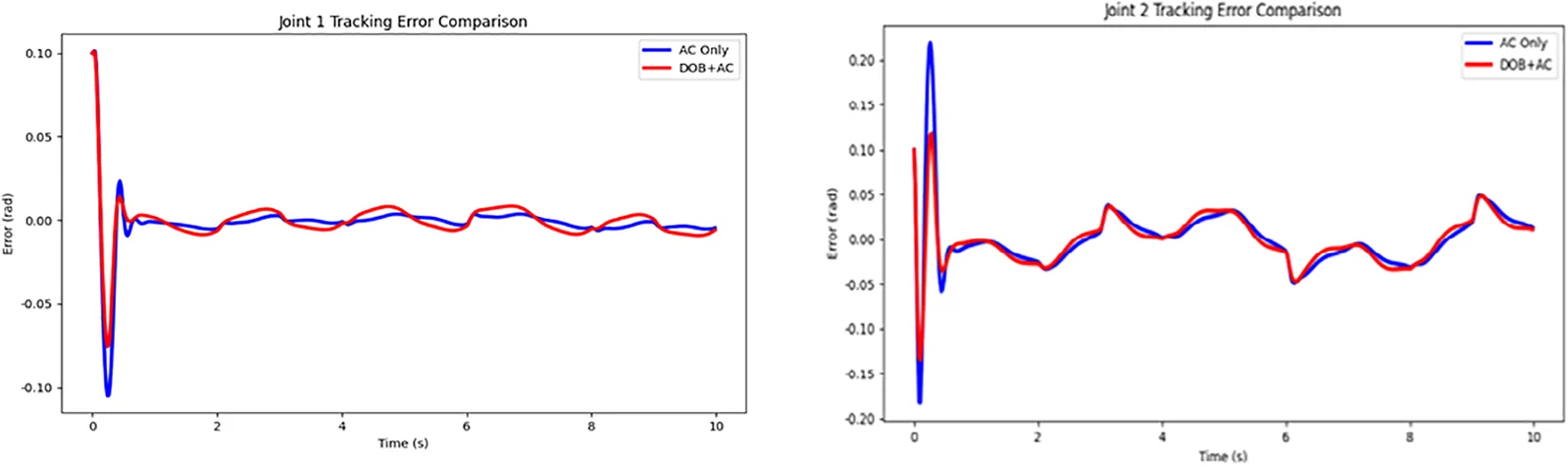
Tracking errors.

The control torques, shown in Fig 5, remain within feasible limits of approximately ±50 Nm for Joint 1 and ±15 Nm for Joint 2. The control signals are smooth and do not exhibit high-frequency chattering, which is a critical advantage for practical implementation and actuator longevity.

**Fig 5:**
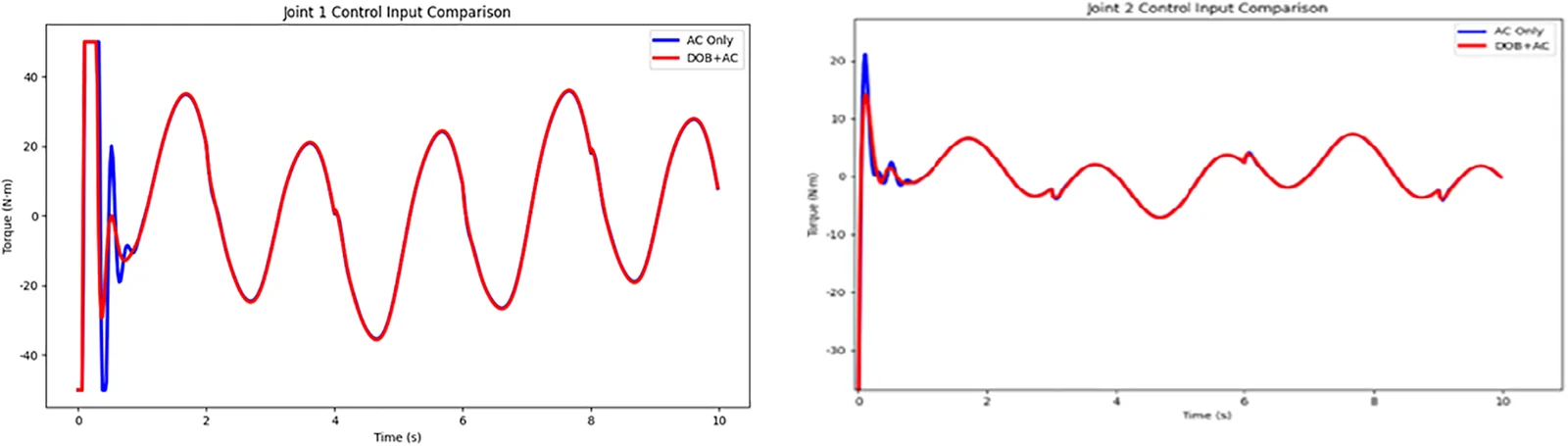
Control torques.

The DOB + AC controller produces smooth, continuously varying torque profiles within ±50 Nm (Joint 1) and ±30 Nm (Joint 2) throughout the motion, whereas the AC Only configuration generates high-frequency torque spikes during the initial transient before collapsing to near-zero output, indicating that without DOB compensation the actor alone is insufficient to sustain adequate disturbance rejection over the full operating period.

### 5.3. Simulation results for scenario 2 with 6-DOF manipulator model

To further validate scalability and robustness, we conducted simulations on the Mitsubishi RV-2FBD 6-DOF industrial manipulator, demonstrating that the proposed controller can handle strongly coupled, high-dimensional nonlinear dynamics with uncertainties. Fig 6 shows that the end-effector trajectory closely tracks the reference in the planar workspace as well as in the x(t) and y(t) components, with a small position error after a brief transient. Fig 7,Fig 8 confirm accurate joint-space tracking: all six joint trajectories follow their references and the tracking errors rapidly converge to a small neighborhood around zero and remain uniformly bounded, supporting the theoretical stability claims. Fig 9 indicates smooth torque profiles within practical limits, and Fig 10 presents stable online training curves, implying effective policy and value-function adaptation. Overall, these results verify that the proposed framework scales effectively to a 6-DOF industrial manipulator and is promising for real-time deployment and extension to other multi-DOF robots.

**Fig 6:**
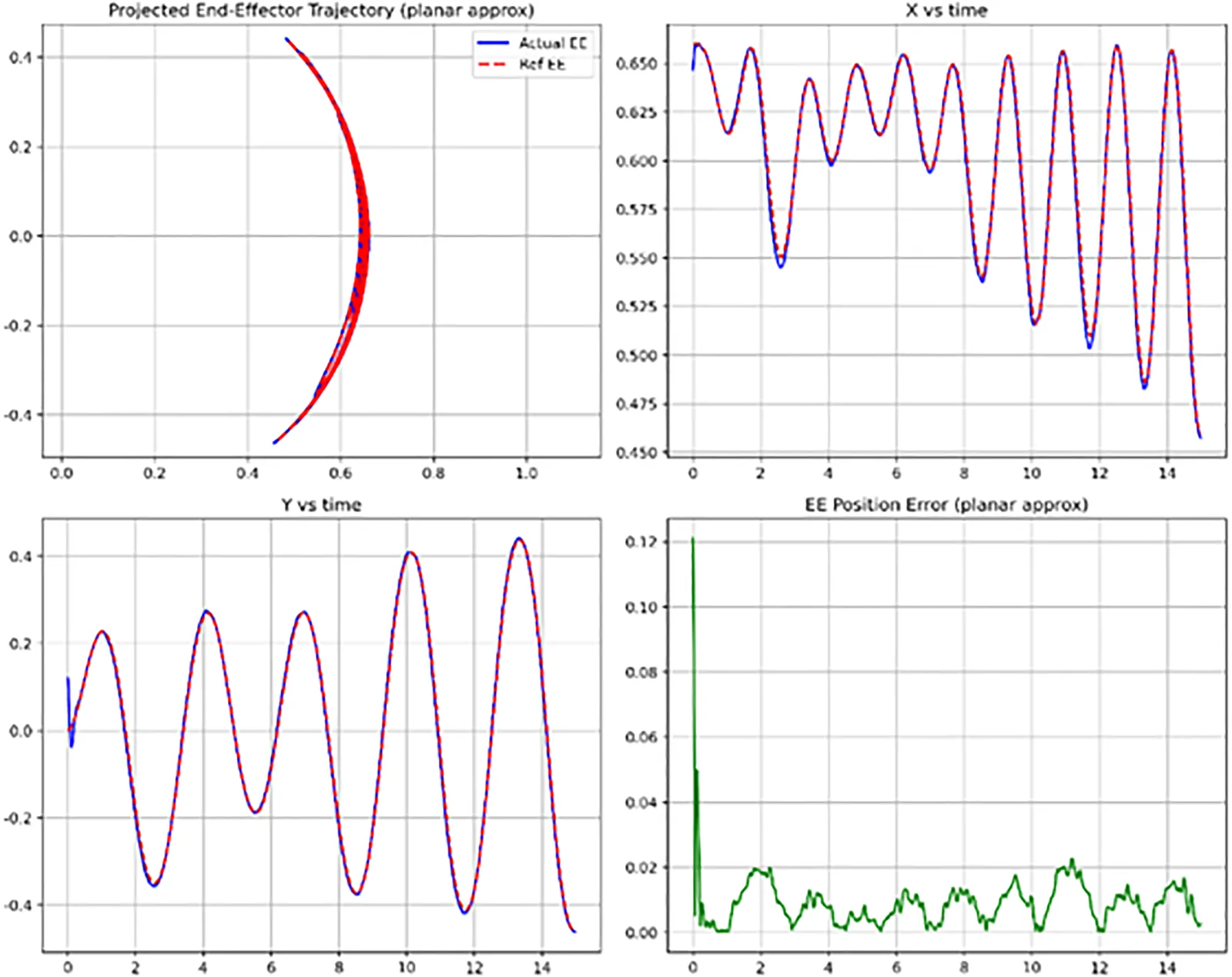
Planar End-Effector trajectory tracking performance.

**Fig 7:**
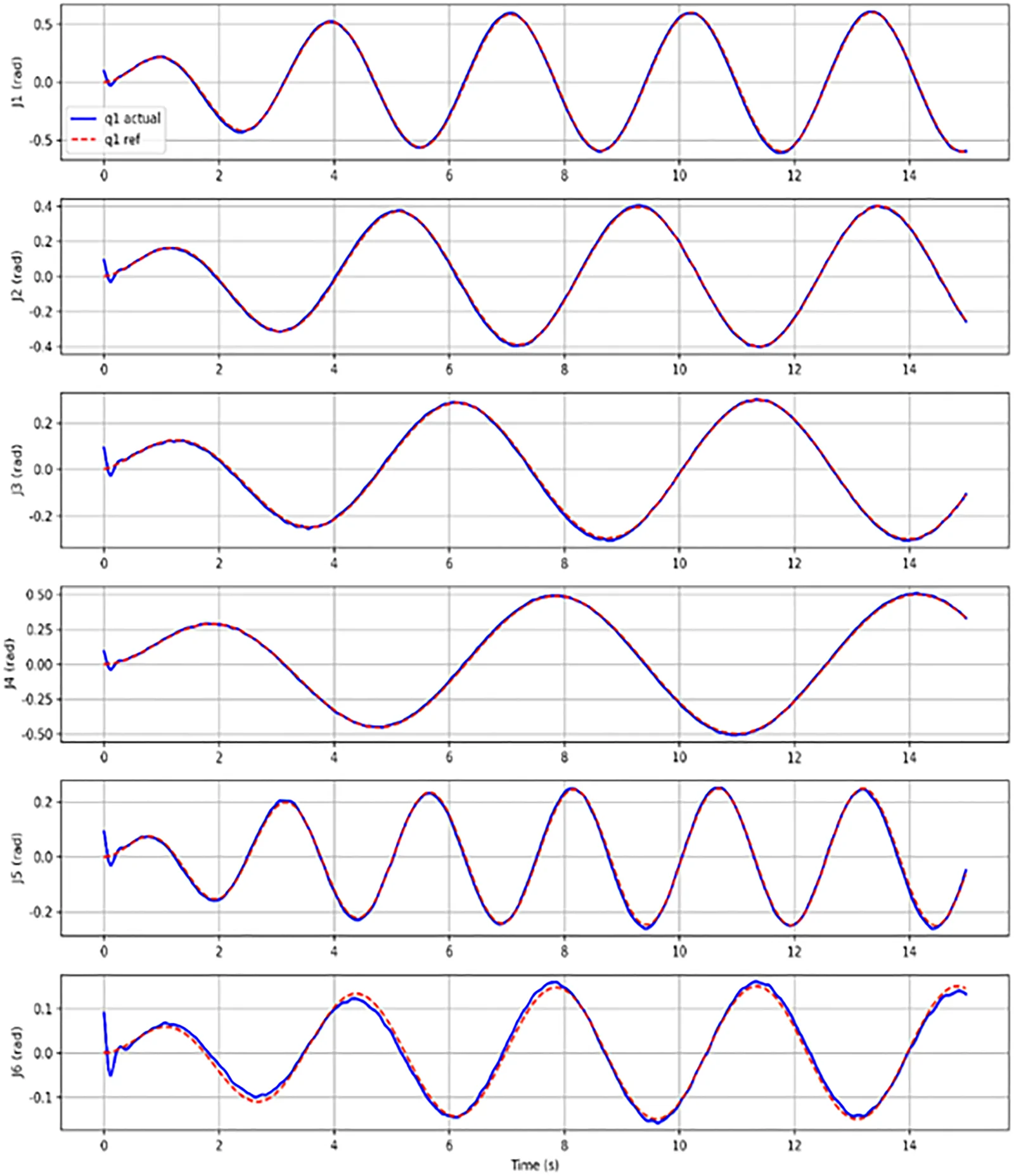
Joint trajectory tracking.

**Fig 8:**
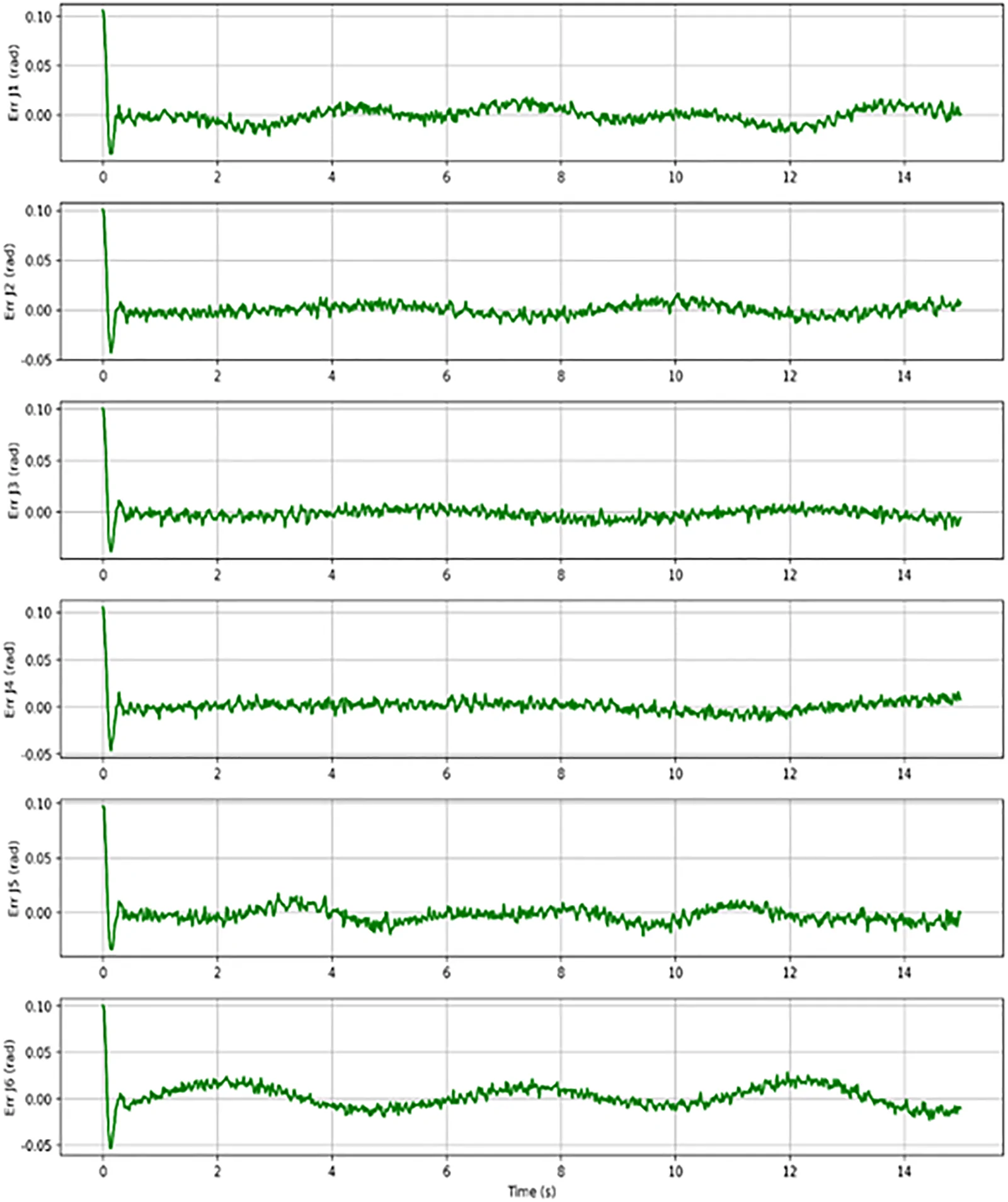
Tracking errors.

**Fig 9:**
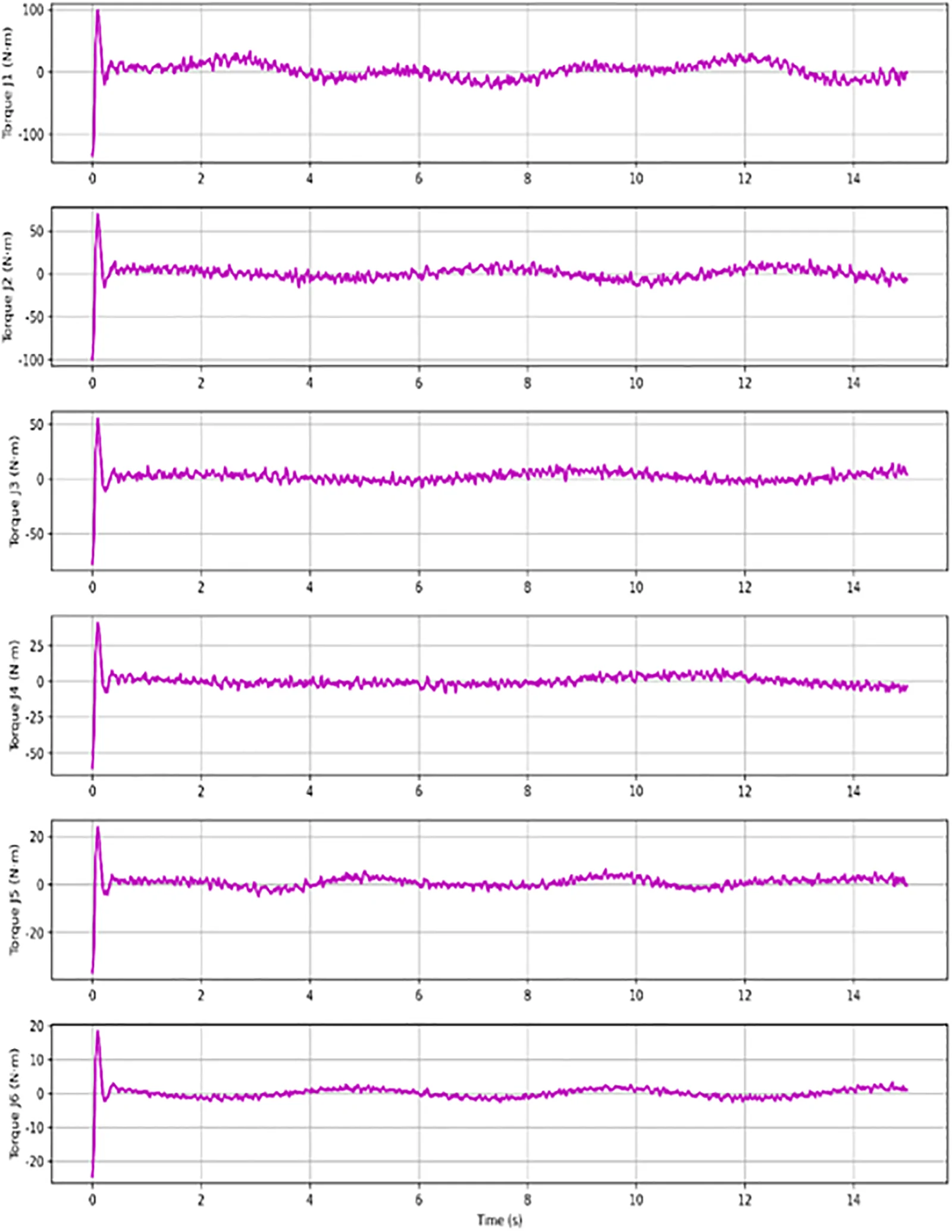
Control torques.

**Fig 10:**
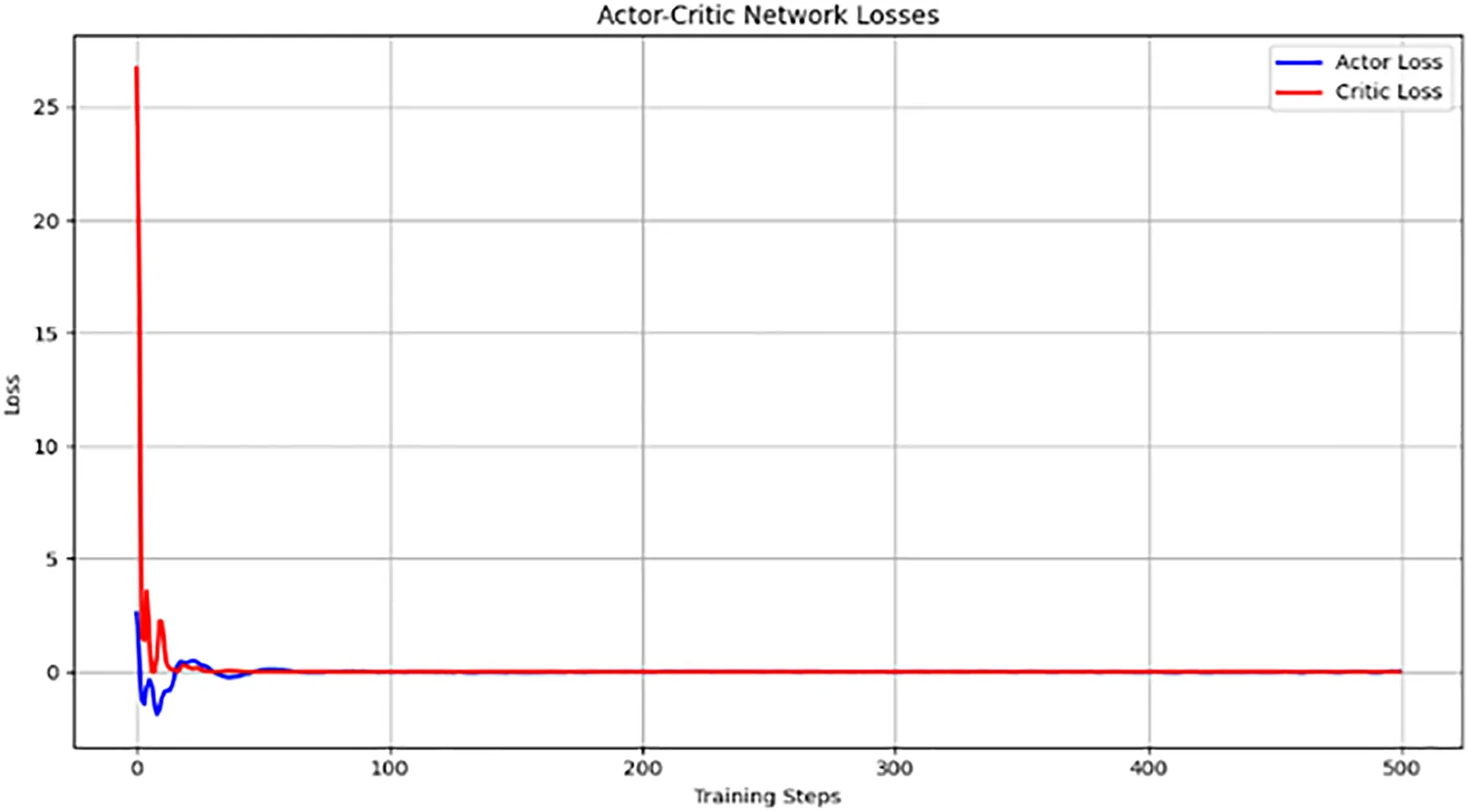
Training steps.

The simulation results for the 6-DOF case collectively demonstrate the scalability and robustness of the proposed DACRL framework under strongly coupled, high-dimensional nonlinear dynamics. In the Cartesian space (Fig 6), the end-effector trajectory closely follows the reference path in both X and Y components, with the position error rapidly decreasing from a peak of approximately 0.12 m at the initial transient to a bounded steady-state band of 0.008–0.02 m after t ≈ 0.5 s, confirming effective task-space regulation despite the increased kinematic complexity. At the joint level (Fig 7), all six joints accurately track their sinusoidal references throughout the 15 s simulation, with Joint 6 exhibiting the largest visible deviation due to its distal position and higher sensitivity to cumulative kinematic coupling. Correspondingly, Fig 8 shows that all joint tracking errors converge within approximately 0.2–0.5 s to a uniformly bounded steady-state band within ±0.02 rad for Joints 1–5 and ±0.03 rad for Joint 6, consistent with the UUB stability guarantees established in the Lyapunov analysis. The control torques (Fig 9) remain within physically feasible bounds across all joints peaking at approximately 100 Nm for Joint 1 during the initial transient and settling to smooth steady-state profiles within ±20 Nm with no sustained high-frequency chattering, confirming that the smooth sliding saturation term in the composite torque law effectively prevents actuator excitation. Finally, Fig 10 shows that both actor and critic losses decrease rapidly within the first 50 training steps and remain bounded near zero thereafter, providing empirical evidence of stable online adaptation under the linear-in-weights parameterization, consistent with the bounded weight assumptions of the theoretical analysis.

### 5.4. Discussion

The online learning process is illustrated in Fig 10. The critic loss decreases rapidly from a peak of approximately 27 within the first 10 training steps and converges to near-zero by step 30, while the actor loss stabilizes near zero by step 20, both remaining stably bounded throughout the remaining 470 steps. This rapid convergence confirms that the disturbance-aware learning state and residual-penalized cost provide sufficiently informative gradient signals for online adaptation within the 1 ms sampling constraint, while the projection-based update mechanism effectively prevents parameter drift under the injected time-varying disturbances. These results should be interpreted as empirical evidence of stable online adaptation under the tested scenario rather than proof of global optimality over the entire state space.

Fig 11 reports the actor and critic losses during online learning. Both losses decrease rapidly and remain bounded, indicating stable numerical learning behaviour under the selected hyperparameters. Loss convergence is interpreted as an empirical signal of learning progress rather than evidence of global optimality.

**Fig 11:**
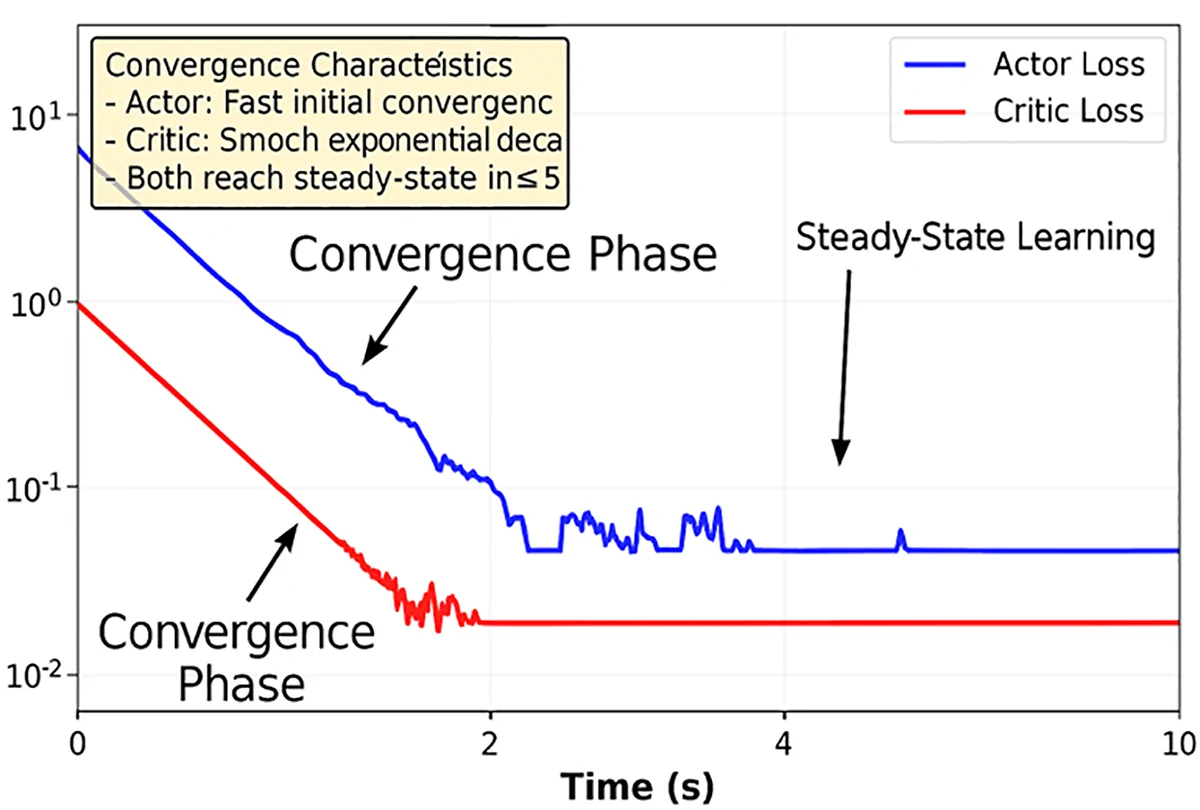
Learning convergence.

Fig 12 shows that the DOB estimate quickly tracks the injected time-varying disturbance and maintains a bounded estimation error, confirming effective disturbance compensation and reduced learning burden. The frequency-domain response indicates disturbance attenuation over the experiment’s bandwidth, reflecting the usual speed–noise trade-off. Table 5 provides a quantitative comparison using MSE of joint tracking. The proposed DACRL achieves the lowest MSE for both joints (Joint 1: 1.25 × 10 ^−^ ⁴ rad²; Joint 2: 4.12 × 10 ^−^ ^4^ rad^2^) and improves upon OADP+DOB [13] under the reported settings.

**Table 5:** Comparative performance analysis MSE.

Control method	Joint 1 MSE (×10 ^−^ ^4^ rad^2^)	Joint 2 MSE (×10 ^−^ ^4^ rad^2^)
DOB-only	8.92	22.45
Standard AC	5.61	15.83
OADP+DOB [13]	3.78	9.45
**Proposed DACRL**	**1.25**	**4.12**

**Fig 12:**
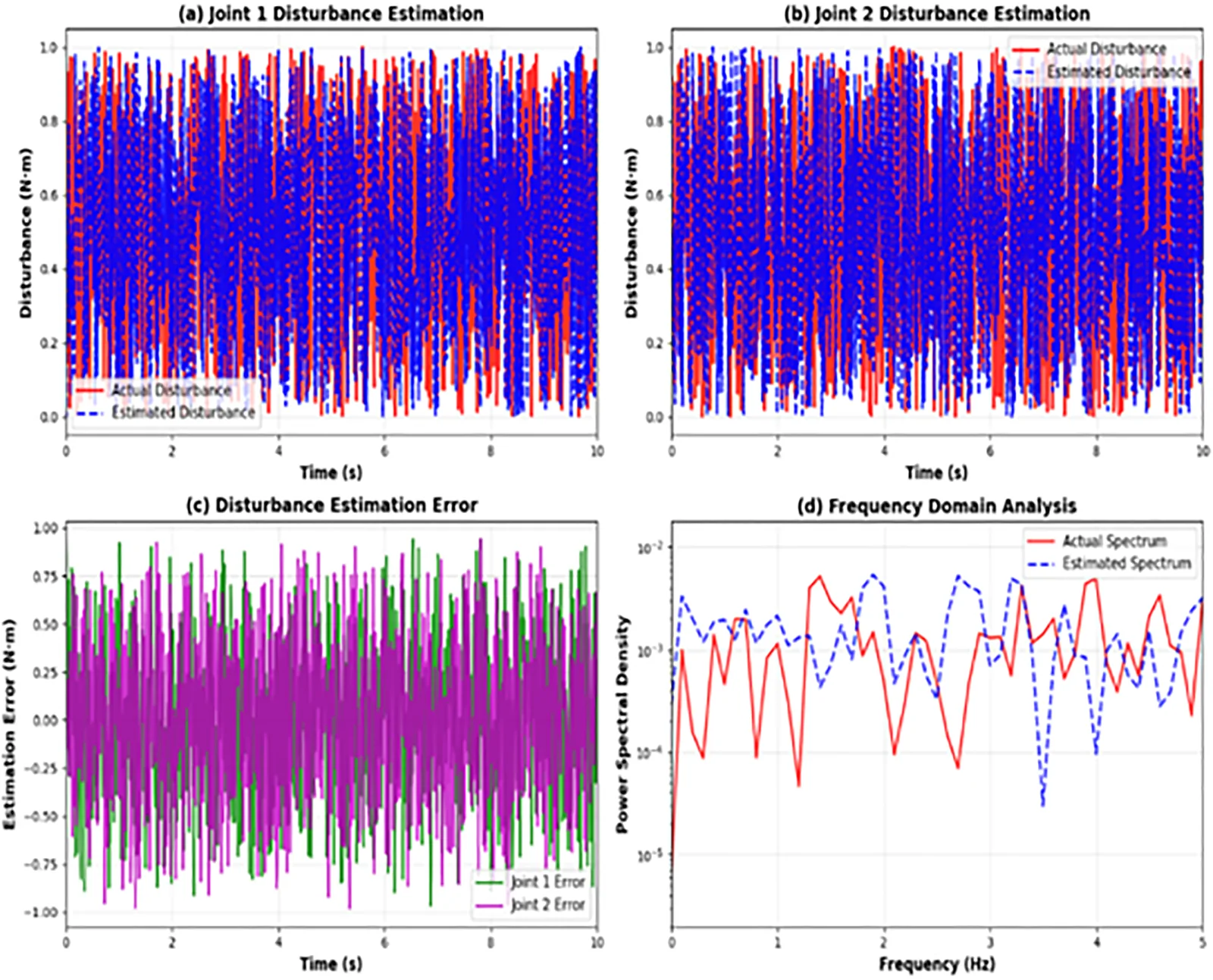
DOB estimation performance.

Table 6 details the steady-state tracking errors and control effort for the 6-DOF case after the initial transient phase.

**Table 6:** Post-transient control performance for the 6-DOF case.

Performance aspect	Metric (post-transient window t∈[0.5,s])	Scholarly interpretation
End-effector tracking accuracy	EE position error: peak ≈ 0.02–0.03; steady-state band ≈ 0.008–0.015	High end-effector tracking accuracy with bounded residual error, indicative of stable closed-loop regulation after transients.
Joint-space peak tracking error	${max}_{i}$	$\begin{array}{c} e_{i}\left(t\right) \end{array}$
Joint-space RMS tracking error	${max}_{i}\text{RMS}\left(e_{i}\right))\approx 0.018\text{\textbackslash{}hspace\{0.17em}\}rad$ (worst joint ≈ J6: 0.012–0.018 rad); others ≈ 0.004–0.012 rad	Low RMS errors support strong steady-state precision; residual bias/low-frequency error is most evident at joint 6.
Transient attenuation	Errors enter the post-transient band within ≈ 0.2–0.5s	Fast transient suppression and absence of drift are consistent with robustness under the tested disturbance profile.
Control effort	Typical steady-state torque magnitudes ≈ 5–40 Nm across joints; initial peak up to ≈ + 100 Nm (J1)	Control inputs remain bounded with relatively smooth steady-state profiles; aggressive initial actuation suggests a trade-off between rapid transient correction and peak effort.
Actuation energy	$\int_{0.5}^{T}|\tau \left(t\right){|}_{\text{2}}^{\text{2}}dt$ dominated by $J_{\text{1}}\approx 9.4kNm^{\text{2}}s$ remaining joints ≈ 0.4k–1.5k	Bounded control energy; should be recomputed from logged time-series for reproducible reporting and fair comparison across baselines.
Constraint compliance	Not explicitly reported (requires $\tau_{max}$ and saturation model)	Hardware relevance requires reporting actuator limits and verifying saturation avoidance; recommended as a required reproducibility item.
Online learning stability	Critic loss peak ≈ 27 then → near-zero within ≈ 50 steps; actor loss → near-zero shortly after	Stable learning dynamics without divergence, suggesting numerically well-conditioned online updates under the tested scenario.

Table 7 further indicates that co-designing the DOB with the learning loop via disturbance-aware state augmentation and objective shaping offers a plausible explanation for improved tracking and smoother control compared with sequential combinations.

**Table 7:** Performance comparison for ablation study.

Configuration	RMSE (rad)	Peak Error (rad)	Torque Energy (10^3^ J)
Robust + DOB (No RL)	0.125 ± 0.012	0.245	4.82
Standard AC $\lambda =0,\text{no}\hat{𝐝}$	0.085 ± 0.009	0.192	5.15
AC + $\hat{𝐝}$ state only λ=0	0.042 ± 0.005	0.098	4.98
**Full DACRL (Proposed)**	**0.018** ± **0.002**	**0.035**	**4.55**

The simulations used fixed controller and learning parameters to ensure reproducibility and fair comparison among the baselines. However, DACRL performance may be affected by several key hyperparameters, including the dob gain, actor/critic learning rates, disturbance penalty, robust gain, and projection bounds. These parameters influence disturbance-estimation speed, noise sensitivity, learning convergence, torque smoothness, and tracking accuracy. therefore, a systematic sensitivity analysis is planned for future work. The main parameters, nominal values, suggested test ranges, and expected effects are summarized in Table 8.

**Table 8:** Planned hyperparameter sensitivity analysis.

Parameter	Nominal value	Tested range	Expected effect
DOB gain α	20	10–40	Larger: faster estimation, more noise sensitivity
Actor learning rate $\eta_{a}$	$1\times 10^{-3}$	$0.5-2\times 10^{-3}$	Larger: faster adaptation, possible oscillation
Critic learning rate $\eta_{c}$	$5\times 10^{-4}$	$2.5-10\times 10^{-4}$	Affects TD-error convergence
Disturbance penalty λ	0.5	0–1.0	Larger: stronger DOB-mismatch compensation
Robust gain $k_{n}$	10	5–20	Larger: better transient rejection, higher torque demand

To quantify the contribution of each disturbance-aware component, we performed an ablation study comparing the full DACRL (state augmentation  +  disturbance-aware cost) with three baselines: (i) robust+dob without rl, (ii) standard actor–critic without disturbance awareness, and (iii) actor–critic with disturbance-aware state only (using d ^ as input but not penalizing it in the cost). All variants used identical network architectures, learning rates, and disturbance profiles. as summarized in Table 3 the full dacrl achieved the lowest rms tracking error and the most efficient torque-energy usage, while the disturbance-aware cost term notably reduced transient oscillations versus state-only augmentation, confirming the benefit of the proposed co-design. The current implementation uses a first-order dob, linear-in-weights actor–critic approximators, and projection-based gradient updates to reduce computational complexity and support real-time control. nevertheless, the computational cost increases with the number of dofs, the size of the disturbance-aware state, and the number of actor/critic basis functions. For higher-dof manipulators, execution time per sampling step, memory usage, and real-time feasibility should be explicitly evaluated. Future work will investigate sparse features, reduced-order basis functions, parallel dob updates, and optimized implementation for high-dimensional robotic systems.

**Limitations and experimental validation.** Although the proposed DACRL framework shows robust tracking performance in both 2-DOF and 6-DOF simulations, the present study is limited to numerical validation. Therefore, the results should be interpreted as a simulation-based proof-of-concept rather than full evidence of real-world deployment readiness. Practical factors such as sensor noise, communication delay, actuator saturation, friction uncertainty, payload variation, and real-time computational constraints may affect closed-loop performance. Future work will validate the proposed controller on a physical manipulator, including payload-change tests, external disturbance rejection tests, trajectory generalization, and comparisons with DOB-only and conventional actor–critic controllers under identical hardware conditions. Safety supervision, including torque limits and possibly control-barrier-function-based constraints, will also be considered before online learning is deployed on hardware.

The present stability analysis assumes bounded and piecewise differentiable matched disturbances. This assumption is standard in many DOB-based robust control designs, but it does not fully cover abrupt impacts, discontinuous disturbances, severe actuator saturation, or actuator faults such as bias, partial loss of effectiveness, or stuck actuators. Although the simulations include sinusoidal disturbances, periodic step changes, and amplitude variations, more aggressive non-smooth disturbances and fault scenarios require additional fault-tolerant and safety mechanisms. Future work will extend the proposed DACRL framework by incorporating fault-detection and isolation, actuator-saturation handling, adaptive fault-tolerant compensation, and safety constraints such as control barrier functions.

## 6. Conclusion and future work

This paper presents DACRL, a co-designed reinforcement learning framework for high-precision robotic trajectory tracking under nonlinear dynamics and disturbances. By integrating a Nonlinear Disturbance Observer (NDOB) directly into the actor–critic learning loop via state augmentation and objective shaping, the system enables real-time disturbance rejection and explicit reaction to uncertainties. Supported by a Lyapunov-based stability analysis ensuring UUB convergence, the method was validated on 2-DOF and 6-DOF (Mitsubishi RV-2FBD) manipulators. Simulation results demonstrated improved tracking accuracy, faster convergence, and smoother torque profiles compared with standard RL and cascade designs under the tested disturbance profiles. However, experimental validation on a physical manipulator is still required to assess real-time implementation issues, actuator constraints, sensor noise, and sim-to-real transfer performance. The current DACRL framework does not explicitly enforce actuator limits or formal safety constraints. Although the simulated torques remain bounded and relatively smooth, practical deployment requires constraints on joint positions, velocities, torques, and collision avoidance to be incorporated into the controller. Future work will integrate actuator saturation handling, safety filters, and control-barrier-function-based constraints to ensure safe online learning and hardware implementation.

## Supporting information

S1 File Simulation source code for the disturbance-aware actor–critic reinforcement learning framework. https://github.com/hung83ddt-glitch/PONE-D-26-05825R2-Plosone. The “**S1 File.zip”** archive contains the Python source code used to perform the 2-DOF and 6-DOF robotic-manipulator simulations.
(ZIP)
